# Cancer incidence inconsistency between UK Biobank participants and the population: a prospective cohort study

**DOI:** 10.1186/s12916-025-03998-z

**Published:** 2025-03-26

**Authors:** Chenxi Li, Gillian S. Dite, Tuong L. Nguyen, John L. Hopper, Shuai Li

**Affiliations:** 1 Centre for Epidemiology and Biostatistics, Melbourne School of Population and Global Health, The University of Melbourne, 207 Bouverie Street, Carlton, VIC 3053 Australia; 2 Children’s Hospital Westmead Clinical School, The University of Sydney, 1 King Street, Newtown, NSW 2042 Australia

**Keywords:** UK Biobank, Cancer incidence, Absolute risk, Cohort study, Standardised incidence ratio, Healthy volunteer bias

## Abstract

**Background:**

While the UK Biobank has been widely used for cancer research, its representativeness of the population in terms of cancer incidence has not been thoroughly investigated.

**Methods:**

We conducted a prospective cohort study of 466,163 UK Biobank participants who were cancer-free at recruitment. Standardised incidence ratios (SIRs) were calculated for all cancers combined and for 25 cancers, by comparing incidences for the participants with the UK national incidences. Variations in SIR by age, sex and deprivation measures were investigated.

**Results:**

Over a median follow-up period of 12 years, 47,535 participants had a cancer diagnosis. The SIR for all cancers combined was 0.90 (95% CI: 0.89, 0.91). The SIR increased with age and deprivation (*P* = 10^−9^). The SIRs of 17 cancers differed from 1 (Bonferroni-adjusted *P* < 0.05): for prostate cancer and melanoma the SIRs were 1.2 and for the other 15 cancers the SIRs ranged from 0.43 to 0.93. The SIRs of 13 cancers differed by deprivation: the greater the deprivation, the lower the SIRs for prostate cancer and melanoma, and the higher the SIRs for the other 11 cancers.

**Conclusions:**

The overall cancer incidence was 10% lower for the UK Biobank participants compared with the population, with most cancers having a lower incidence that increased with deprivation. Irrespective of their causes, the inconsistencies could bias UK Biobank research results related to absolute cancer risks, such as the development and/or validation of cancer risk models and penetrance estimates for cancer susceptibility genes.

**Supplementary Information:**

The online version contains supplementary material available at 10.1186/s12916-025-03998-z.

## Background

The UK Biobank is one of the world’s leading biobank cohorts which has recruited about 500,000 participants from 2006 to 2010 and followed them up for clinical outcomes through linkage to medical records, and national cancer and death registries [1]. This resource has been widely used for medical and health research, especially for cancer research.

One type of cancer research using the UK Biobank resource is to develop and/or validate cancer risk models, such as those for lung [2–4], colorectal [5, 6], prostate [7], pancreatic [8, 9], breast [10] and kidney cancers [11]. While most of these models reported good discrimination of cancer risk (i.e. to differentiate whether a person has cancer or not), the calibration of the models in a population setting (i.e. the agreement between the actual and estimated risk) relies on the cancer incidences for UK Biobank participants being similar to those for the population. Differential participation on the basis of cancer incidence would bias the calibration results; for example, a risk model with a good prediction for the population would appear to underpredict risk when the calibration was conducted using a sample with a higher cancer incidence than the population, as has been observed by a UK Biobank study of the prostate cancer risk model CanRisk-Prostate [7].

Studies have reported that UK Biobank participants have different cancer incidences compared with the population: the incidence of any cancer for participants aged 70–74 years was about 11.8% lower for males and 18.1% lower for females [12]; 7% fewer observed breast cancers than those predicated based on population incidence over 5 years of follow-up, with variations by age groups [10]; lower incidences of colorectal, endometrial, lung and kidney cancers over an average of 5.5 years of follow-up [12]; and a higher incidence of prostate cancer over 5 or 10 years of follow-up [7, 12]. Whether the incidences of other cancers are consistent with the population incidences is unclear. These studies investigated cancer incidence during a follow-up time of only 5–10 years. It is unclear whether the inconsistency in cancer incidence exists for a longer follow-up time.

To guide the use of the UK Biobank in cancer research, we compared the cancer incidence for the UK Biobank participants with the population incidence, for cancers overall and 25 cancers specifically, and assessed whether the incidence inconsistency varied by participant demographic and socioeconomic characteristics.

## Methods

### Study sample

Between 2006 and 2010, around 9.2 million NHS registers residing within 25 miles of one of 22 assessment centres in Scotland, England and Wales were invited to join the UK Biobank. In total, about 502,000 participants (5.5% of those invited) were recruited, covering a variety of different settings for socioeconomic and ethnic heterogeneity and urban-rural mix [1]. All recruited participants received a baseline assessment at a centre to provide comprehensive information about their health and lifestyle, including written consent, touch screen questionnaires for detailed diet recall, face-to-face interviews with a study nurse, physical measurement, and sample collection of blood, urine and saliva.

We excluded participants who had any cancer diagnosed before or at the recruitment, withdrew or had an age at recruitment < 0 in the database, resulting in 466,163 participants. The UK Biobank has approvals from the North West Multi-centre Research Ethics Committee as a Research Tissue Bank approval. All participants gave consent for their de-identified data to be used for health-related research that is in the public interest.

### Cancer diagnosis and death data

Cancer diagnosis including cancer type and age at diagnosis was determined using self-reported cancer diagnosis, linked national cancer registry data (in ICD-9 and ICD-10 codes) and linked causes of death to national death registries (in ICD-10 code). Age at death was determined using the linked death registry data.

We investigated all cancer diagnoses excluding non-melanoma skin cancer, as well as 25 major types including cancers in the bladder, bone, brain and central nervous system, breast, cervix uteri, colorectum, corpus uteri, eye, gallbladder, head and neck, kidney, liver, lung, oesophagus, ovary, pancreas, prostate, soft and connective tissue, stomach, testis and thyroid, and leukaemia, lymphoma, melanoma, and multiple myeloma. The diagnosis codes used for identifying these cancers are in Additional file 1: Table S1.

### Participant characteristics data

Participant characteristics analysed in this study included date of birth, sex, age at baseline, assessment centre, date lost to follow-up, index of multiple deprivation (field IDs: 26410, 26426, 26427) (a greater value reflecting greater deprivation) and average total household income before tax (field ID: 738). The index of multiple deprivation is a score combining the deprivation scores of several subdomains (e.g. crime score, education score, housing score) at the small area level, and it reflects the deprivation experienced by the people living in the area. The index is calculated using different subdomains for participants in England, Scotland and Wales; see the notes of category 76 on UK Biobank’s showcase for more details https://biobank.ndph.ox.ac.uk/showcase/label.cgi?id=76.

### Population cancer incidence data

The UK nationally representative cancer incidence data from 1998 to 2017 were obtained through the publicly available data of the Office of National Statistics. The data are based on registrations of primary malignant neoplasm diagnoses in England, the same ascertainment method for the vast majority (98.3%) of the UK Biobank incident cancer diagnoses in this study. In this study, the cancer incidences after 2017 were assumed to be the same as those in 2017. The data were used to derive birth year-, sex- and age-specific population cancer incidences.

### Statistical analysis

Participants were followed up from the baseline at recruitment to the first cancer diagnosis, death, lost follow-up or linked cancer data censoring date, whichever was first. The linked cancer data censoring date was 31 December 2020, 31 December 2016 and 30 November 2021 for those residing in England, Wales and Scotland, respectively. The participants were assumed to reside in the relevant country based on the location of the recruitment assessment centre. For a cancer of interest, a participant was considered affected if they were diagnosed with that cancer during the follow-up, otherwise unaffected.

For each participant, the probability of developing the cancer of interest during the follow-up was calculated as

$$\text{Pr}=1-\prod_{i=m}^{n-1}{e}^{{-r}_{i}}$$

where *m* is the baseline age, *n* is the age at the end of follow-up and *r* is the birth year- and sex-specific population incidence at age *i* (in years). The expected number of cancer cases in the study sample is equal to the sum of the probabilities of all the participants.

The consistency between the observed and expected numbers of cancer cases was investigated using the standard incidence ratio (SIR), defined as the observed number divided by the expected number. The UK Biobank incidence is higher than the population incidence if the SIR > 1. Poisson regression was used to calculate the SIR for the whole study sample, as well as by sex, age groups (< 45, 45–49, 50–54, 55–59, 60–64, ≥ 65 years), quartiles of index of multiple deprivation and household income (< £18,000, £18,000–£30,999, £31,000–£51,999, ≥ £52,000). The difference between by sex was tested using the likelihood ratio test, and the difference by age, deprivation and household income was tested by fitting a linear model with each subgroup taking the median value of the subgroup. Due to missing data in the index of multiple deprivation and household income, 578 and 70,668 participants, respectively, were excluded from the subgroup analysis of these variables.

The analyses were conducted using R (version 4.2.2). All statistical tests were two-sided. Bonferroni adjustment was used to adjust for the multiple testing across 25 cancers.

## Results

The 466,163 participants included had a median baseline age of 57 years (range: 37–73 years). Over a median follow-up time of 12 years (range: 1–15 years), 47,535 participants had a cancer diagnosis (22,029 females and 25,506 males) (Table 1). Males had a higher cancer incidence than females (*P* = 10^−15^). The cancer incidence increased with age (*P* = 10^−15^) and decreased with increasing household income (*P* = 10^−15^); there was no substantial difference between the quartiles of the index of multiple deprivation (*P* = 0.69).

**Table 1:** The incidence of all cancers combined for the UK Biobank participants

	Number of participants	Person year	Number of cancers	Cancer incidence (per 100,000 person-years)
By age group				
<45 years	49,773	579,684	1783	307.6
45–49 years	63,609	734,474	3165	430.9
50–54 years	72,127	823,947	5039	611.6
55–59 years	84,479	947,701	8441	890.7
60–64 years	110,475	1,205,380	14,775	1225.8
≥65 years	85,700	907,528	14,332	1579.2
By sex				
Females	250,281	2,820,711	22,029	781.0
Males	215,882	2,378,003	25,506	1072.6
By index of multiple deprivation				
First quartile (<−3.64)	116,365	1,305,926	11,977	917.1
Second quartile (−3.64 to −2.12)	116,378	1,299,262	12,026	925.6
Third quartile (−2.13, 0.551)	116,443	1,295,468	11,571	893.2
Fourth quartile (0.552, 11)	116,399	1,291,571	11,920	922.9
By average total household income before tax				
<£18,000	88,430	963,490	11,089	1150.9
£18,000–£30,999	99,720	1,103,770	11,117	1007.2
£31,000–£51,999	103,900	1,168,413	9493	812.5
≥£52,000	103,445	1,172,835	8185	697.9

Based on the population incidence, the participants were expected to have 52,840 cancers, resulting in a SIR of 0.90 (95% confidence interval [CI]: 0.89, 0.91; Table 2); males had a SIR of 0.95 (95% CI: 0.94, 0.96), higher than the 0.85 (95% CI: 0.84, 0.86) for females (*P* = 10^−33^). The SIR increased with age (*P* = 10^−9^) and the index of multiple deprivation (*P* = 10^−20^) and decreased with the household income (*P* = 10^−20^), with the lowest SIR observed for those who were the least deprived or with the most household income.

**Table 2:** Standard incidence ratios for all cancers combined for the UK Biobank participants

	Observed number of cancers	Expected number of cancers	SIR (95% CI)	P*
Overall	47,535	52,840.0	0.90 (0.89, 0.91)	
By sex				1.8 × 10^−34^
Females	22,029	25,970.4	0.85 (0.84, 0.86)	
Males	25,506	26,869.6	0.95 (0.94, 0.96)	
By age group				
<45 years	1783	1964.6	0.91 (0.87, 0.95)	7.9 × 10^−10^
45–49 years	3165	3652.1	0.87 (0.84, 0.90)	
50–54 years	5039	5974.6	0.84 (0.82, 0.87)	
55–59 years	8441	9750.8	0.87 (0.85, 0.88)	
60–64 years	14,775	16,182.0	0.91 (0.90, 0.93)	
≥65 years	14,332	15,315.8	0.94 (0.92, 0.95)	
By index of multiple deprivation				
First quartile (<−3.64)	11,977	13,838.5	0.87 (0.85, 0.88)	9.1 × 10^−21^
Second quartile (−3.64 to −2.12)	12,026	13,642.4	0.88 (0.87, 0.90)	
Third quartile (−2.13, 0.551)	11,571	13,026.4	0.89 (0.87, 0.90)	
Fourth quartile (0.552, 11)	11,920	12,277.3	0.97 (0.95, 0.99)	
By average total household income				
<£18,000	11,089	11,409.7	0.97 (0.95, 0.99)	2.9 × 10^−21^
£18,000–£30,999	11,117	12,305.7	0.90 (0.89, 0.92)	
£31,000–£51,999	9493	10,891.8	0.87 (0.85, 0.89)	
≥£52,000	8158	9558.5	0.85 (0.84, 0.87)	

^*^Linear trend test *P* values for subgroup analyses by age, deprivation and household income

**Fig. 1:**
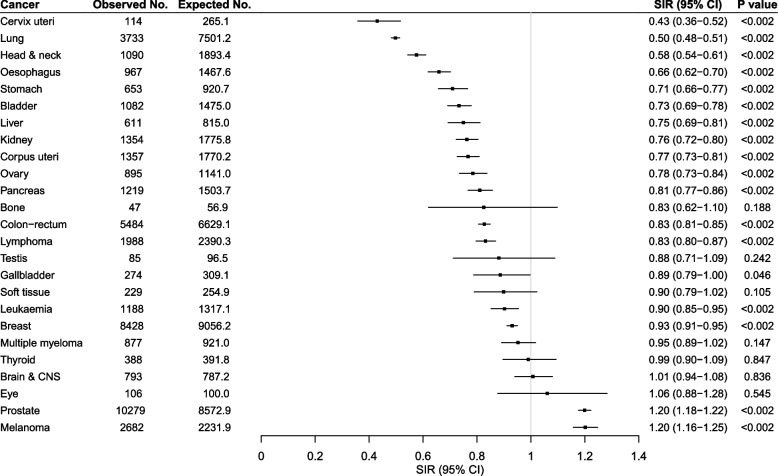
Standardised incidence ratios for 25 cancers for the UK Biobank participants

For individual cancer types, the SIRs of 17 cancers were significantly different from 1 after the Bonferroni adjustment (*P* < 0.05/25 = 0.002; Fig. 1). The SIRs of prostate cancer and melanoma were 1.2, while for the other 15 cancers, their SIRs ranged from 0.43 to 0.93.

**Fig. 2:**
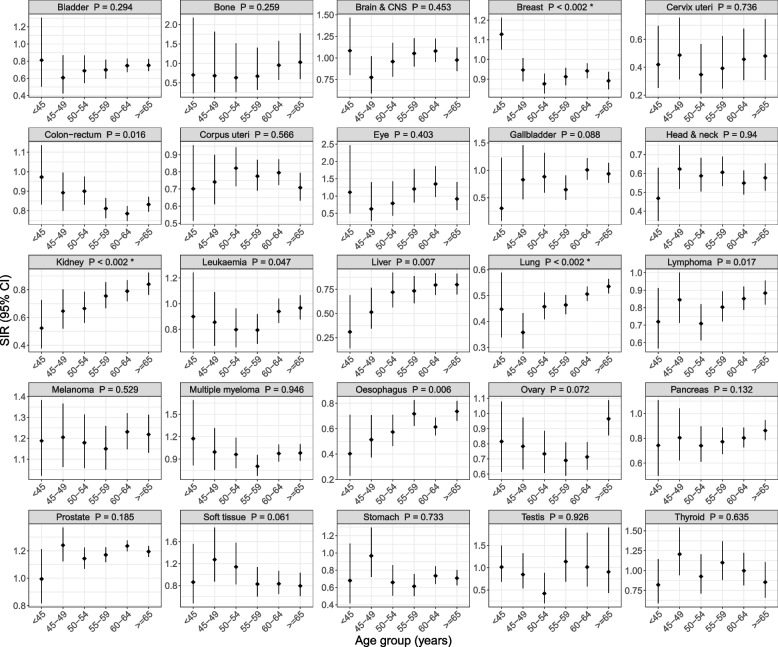
Standardised incidence ratios for 25 cancers by the age at baseline

None of the 20 non-sex-specific cancers had a SIR that differed between females and males (all Bonferroni-adjusted *P* > 0.48; Additional file 1: Fig. S1). The SIRs for three cancers differed with age (all Bonferroni-adjusted *P* < 0.02; Fig. 2). The breast cancer SIR decreased with age from 1.13 (95% CI: 1.05, 1.21) for participants aged < 45 years to SIRs < 1 for older participants. The SIRs for lung and kidney cancers increased with age, though their SIRs were < 1 for all age groups.

**Fig. 3:**
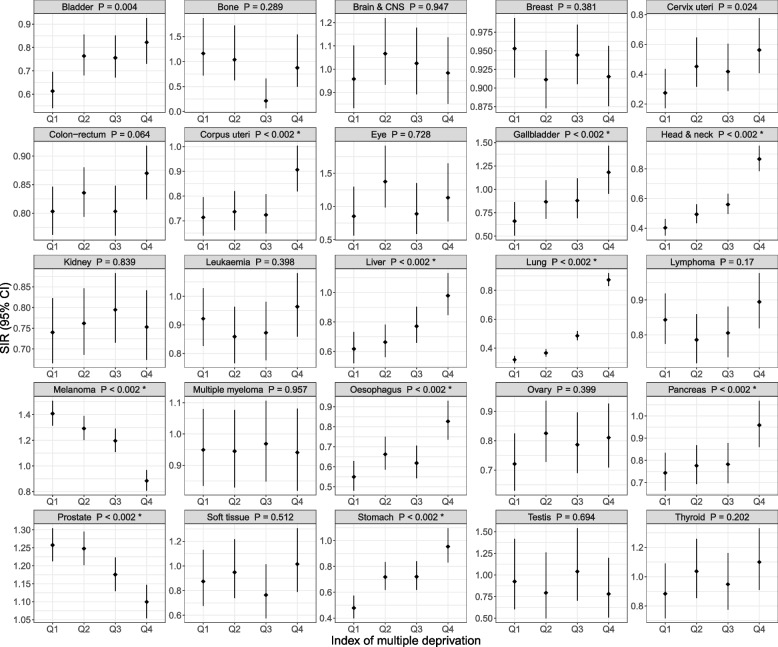
Standardised incidence ratios for 25 cancers by the index of multiple deprivation

The SIRs for 10 cancers differed by the index of multiple deprivation (all Bonferroni-adjusted *P* < 0.02; Fig. 3). The melanoma SIR decreased with deprivation: the participants in the 4th quartile, i.e. the most deprived, had a SIR < 1 (0.88, 95% CI: 0.81, 0.97), while the 1st to 3rd quartiles all had a SIR > 1. The prostate cancer SIR also decreased, though all quartiles had a SIR > 1. For the other eight cancers, their SIRs increased with deprivation.

**Fig. 4:**
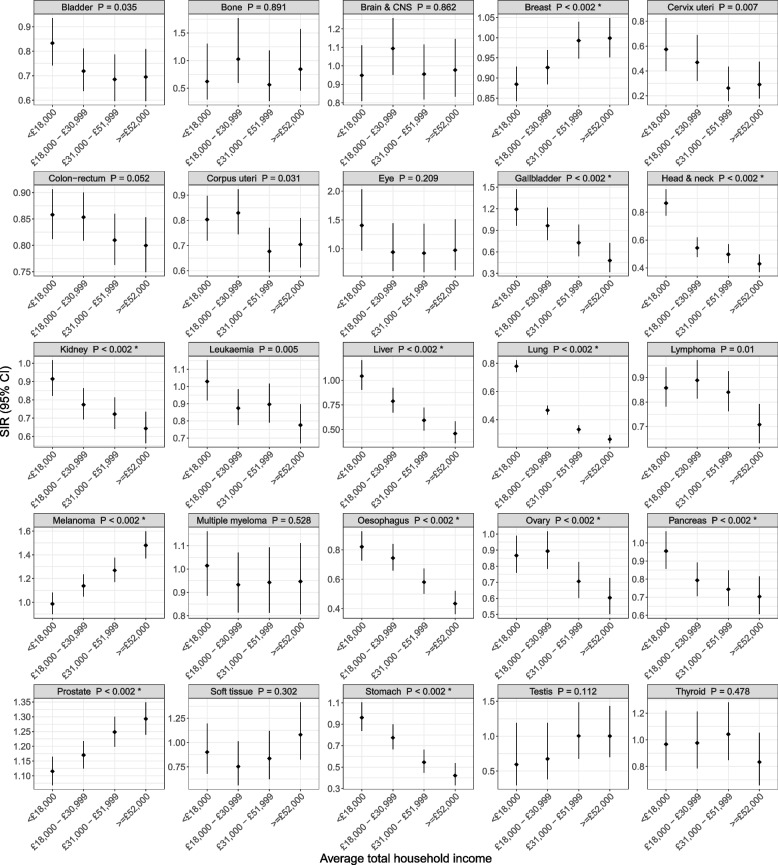
Standardised incidence ratios for 25 cancers by the average household income

The SIRs of 12 cancers differed by household income (all Bonferroni-adjusted *P* < 0.01; Fig. 4). The SIRs of breast cancer, prostate cancer and melanoma increased with household income: with higher income, breast cancer incidence was closer to the population incidence, while prostate cancer and melanoma incidences were higher than the population incidences. For the other nine cancers, their SIRs decreased with the household income.

In summary, the SIRs of 13 cancers differed by deprivation or household income. Additional file 2: Table S2 provides a summary of the results for the 25 cancers from the main and subgroup analyses.

## Discussion

This study assessed whether cancer incidence for participants in the UK Biobank was consistent with those for the population and found that, for cancers overall, it was 10% lower. Inconsistency was also observed for 17 types of cancers, with the majority having a lower incidence for UK Biobank participants: the greatest differences were observed for cervical, lung, head and neck, and oesophageal cancers, of which the incidences were more than 30% lower. While previous studies also compared the cancer incidence for UK Biobank participants with those for the population, they only investigated a few cancers including breast, colorectal, endometrial, kidney, lung and prostate cancers [7, 10, 12]. To our knowledge, this study is the first that investigated 25 major cancers comprehensively.

It has been reported that, for some health-related measures, the UK Biobank has a ‘healthy volunteer’ participation bias [12]: compared with the population, UK Biobank participants have a healthy lifestyle (less likely to be obese, to smoke and to drink alcohol) and fewer self-reported health conditions. Given the known associations between some of these measures and some cancer incidences, this participation bias could lead to the lower cancer incidences observed in the UK Biobank. Lower incidences of some cancers and lower mortality have also been observed for participants in other studies, which are also speculated to be attributed to healthy volunteer bias studies [13–16].

There is evidence that the healthy volunteer bias might attenuate with participants aging and acquiring some chronic conditions [13, 14]. This has been shown by results from three long-standing community-based studies in the USA, suggesting that participants had lower mortality risk in the first 10 years of follow-up compared with non-participants, but no difference was observed after > 30 years of follow-up [17]. Our findings on breast, colorectal, endometrial, kidney and lung cancer based on a median follow-up period of 12 years are consistent with previous findings on these cancers based on a short follow-up period of 5–10 years [7, 10, 12], suggesting the healthy volunteer bias still exists with this a longer period of follow-up.

Lower cancer incidences for the UK Biobank participants were more pronounced for females, younger participants and participants who were less deprived, which is also consistent with the healthy volunteer bias. Females in the UK Biobank on average live a healthier life, as suggested by that they have lower proportions of obesity, current smokers, alcohol drinking and risk-taking behaviours than male participants [12, 18]. The sex difference has also been observed in other studies, such as the standardised mortality ratio and cancer standardised incidence ratios are lower in females than in males in the Prostate, Lung, Colorectal, and Ovarian Cancer Screening Trial [14]. Compared with older participants in the UK Biobank, younger participants are less likely to be obese, smokers or alcohol drinkers [12]. Deprivation and house income could reflect socioeconomic status, and socioeconomic status is positively related to a healthy lifestyle [19].

This study found that the lung cancer SIR increased with age, i.e. an incidence more consistent with the population one was observed for older participants. This could be due to that the older participants have a higher proportion of smokers [12]. Varied proportions of smokers by age could also contribute to the observed kidney cancer SIR increasing with age. Breast cancer SIR was > 1 for participants aged < 45 years and < 1 for those aged ≥ 45 years. Women with pathogenetic variants in high-risk breast cancer susceptible genes such as *BRCA1* and *BRCA2* have a greater breast cancer risk, especially at young ages [20, 21]; however, the proportions of women with such pathogenetic variants are similar between participants in the UK Biobank and the population [21, 22], so that the elevated SIR in ages < 45 years is unlikely to be due to this. These young women might have increased screening.

Our results also suggest that the healthy lifestyle or ‘heath’ per se might not be sufficient to explain the observed differences in cancer incidence, and there must be other factors that play a role, given that not all cancers had a lower incidence—higher incidences were observed for prostate cancer and melanoma. The 20% higher incidence of prostate cancer might be due to that there are greater proportions of health-conscious males who take voluntary prostate-specific antigen testing in the UK Biobank. This hypothesis is supported by our findings that the higher the socioeconomic status, which is related to greater health consciousness [23, 24], the higher the prostate cancer incidence than the population incidence. The higher melanoma incidence could be due to that the UK Biobank participants, who in general are less deprived than the population [12], spend more time outdoors to be exposed to the sun and/or have more frequent mole checks. These hypotheses are supported by the findings that the participants who were less deprived or who had a higher income had an even higher incidence.

Irrespective of their causes, the observed inconsistencies suggest that for most cancers, their *absolute* risks in the UK Biobank are not the same as those in the population. This can bias research findings based on the UK Biobank related to absolute cancer risks, such as (1) cancer risk model development and/or validation: the observed risk overestimates of lung and colorectal cancer risk models and the underestimate of prostate cancer risk model in the UK Biobank could be due to this [3, 6, 7] and (2) the penetrance estimates for cancer susceptibility genes [25]. These types of research should consider the inconsistency and address it, like calibrating the cancer risk in the UK Biobank to agree with those in the population using the SIRs estimated from this study. On the other hand, the biased cumulative cancer risks do not necessarily lead to biased relationships between exposures and cancers, i.e. generalisable *relative* risk estimates could still be achieved in studies as sufficiently large as the UK Biobank [26].

The major strength of this study is that we conducted the most comprehensive analysis for 25 cancers using the most up-to-date data and investigated the consistency by factors including sex, age, deprivation and household income. One limitation is that although the index of multiple deprivation and household income could reflect deprivation to some extent, they might not be able to measure deprivation accurately: the index of multiple deprivation is area-based rather than individual-level, and income does not consider other factors related to deprivation like wealth [27]. Other limitations include as follows: (1) The exclusion of participants who had cancer diagnosed before or at the recruitment resulted in a ‘healthy’ study sample by design, which might contribute to the observed cancer incidence inconsistency; however, most UK Biobank cancer cohort studies use the same exclusion criterion, and using this criterion fits our purpose to guide the use of the UK Biobank in cancer research. (2) UK Biobank cancer data do not have information on subtypes, which limited us from investigating if the cancer incidence inconsistency could be due to different subtype distributions between the UK Biobank participants and the population. (3) We also used self-reported data to determine cancer diagnosis, a different ascertainment method from the population cancer incidence data; however, self-reported cancer diagnoses only accounted for < 2% of the total observed diagnoses, and this proportion is unlikely to substantially impact the results.

## Conclusions

The cancer incidence for UK Biobank participants is not consistent with the population incidence, with most cancers having a lower incidence and prostate cancer and melanoma having a higher incidence. Such disagreements could bias the research findings related to the absolute risk of cancer, and relevant cancer research should take this bias into account.

## Supplementary Information

Additional file 1: Tables S1–S2 and Fig. S1. Fig. S1 Standardised incidence ratios for 20 cancers by sex.

## Data Availability

This research was conducted using the UK Biobank data under application number 47401. Data access requests should be made to the UK Biobank.

## Abbreviations

CI: Confidence interval
ICD: International Classification of Diseases
SIR: Standardised incidence ratio
