# Supplementary material for: Cancer incidence inconsistency between UK Biobank participants and the population: a prospective cohort study
Source: BMC Med. 2025 Mar 26;23:181. doi: 10.1186/s12916-025-03998-z (PMC11948887; doi:10.1186/s12916-025-03998-z)

**Table S1. ICD-9, ICD-10, and self-reported cancer codes for the diagnoses of 25 cancers in the UK Biobank**

| **Cancer** | **ICD-10** | **ICD-9** | **Self-reported cancer code** |
| --- | --- | --- | --- |
| Bladder | C67 | 188 | 1035 |
| Bone | C40-C41 | 170 | 1063 |
| Brain and central nervous system | C70-72 | 191-192 | 1031-1033 |
| Breast | C50 | 174-175 | 1002 |
| Cervix uteri | C53 | 180 | 1041 |
| Colon-rectum | C18-C21 | 153-154 | 1020-1023, 1086 |
| Corpus uteri | C54 | 182 | 1040 |
| Eye | C69 | 190 | 1030, 1075 |
| Gallbladder | C23-24 | 156 | 1025 |
| Head and neck | C00-14, C32 | 140-149, 161 | 1004- 1012, 1015-1016, 1077-1079 |
| Kidney | C64-C65 | 189 | 1034, 1067 |
| Leukaemia | C91-95 | 204-208 | 1048, 1074 |
| Liver | C22 | 155 | 1024 |
| Lung | C33-34 | 162 | 1001, 1027-1028, 1080, 1084 |
| Lymphoma | C81-86, C96 | 200-202 | 1047, 1052-1053 |
| Melanoma | C43 | 172 | 1059 |
| Myeloma | C88, C90 | 203 | 1050 |
| Oesophagus | C15 | 150 | 1017 |
| Ovary | C56 | 183 | 1087 |
| Pancreas | C25 | 157 | 1026,1088 |
| Prostate | C61 | 185 | 1044 |
| Soft tissue | C47, C49 | 171 | 1029, 1068 |
| Stomach | C16 | 151 | 1018 |
| Testis | C62 | 186 | 1045 |
| Thyroid | C73 | 193 | 1065-1066 |

**Table S2. Results summary for the 25 cancers from the main and subgroup analyses**

| Cancer | Overall SIR | Trend by age | Trend by index of deprivation | Trend by household income |
| --- | --- | --- | --- | --- |
| Bladder | ↓ | − | − | − |
| Bone | − | − | − | − |
| Brain and central nervous system | − | − | − | − |
| Breast | ↓ | ↓ | − | ↑ |
| Cervix uteri | ↓ | − | − | − |
| Colon-rectum | ↓ | − | − | − |
| Corpus uteri | ↓ | − | ↑ | − |
| Eye | − | − | − | − |
| Gallbladder | − | − | ↑ | ↓ |
| Head and neck | ↓ | − | ↑ | ↓ |
| Kidney | ↓ | ↑ | − | ↓ |
| Leukaemia | ↓ | − | − | − |
| Liver | ↓ | − | ↑ | ↓ |
| Lung | ↓ | ↑ | ↑ | ↓ |
| Lymphoma | ↓ | − | − | − |
| Melanoma | ↑ | − | ↓ | ↑ |
| Multiple myeloma | − | − | − | − |
| Oesophagus | ↓ | − | ↑ | ↓ |
| Ovary | ↓ | − | − | ↓ |
| Pancreas | ↓ | − | ↑ | ↓ |
| Prostate | ↑ | − | ↓ | ↑ |
| Soft tissue | − | − | − | − |
| Stomach | ↓ | − | ↑ | ↓ |
| Testis | − | − | − | − |
| Thyroid | − | − | − | − |

−: The overall SIR was no different from 1, or there was no evidence of trend by age, index of deprivation or household income

↑: The overall SIR was greater than 1, or there was evidence that the SIR increased with age, index of deprivation or household income

↓: The overall SIR was smaller than 1, or there was evidence that the SIR decreased with age, index of deprivation or household income

**Figure S1. Standardised incidence ratios for 20 cancers by sex**


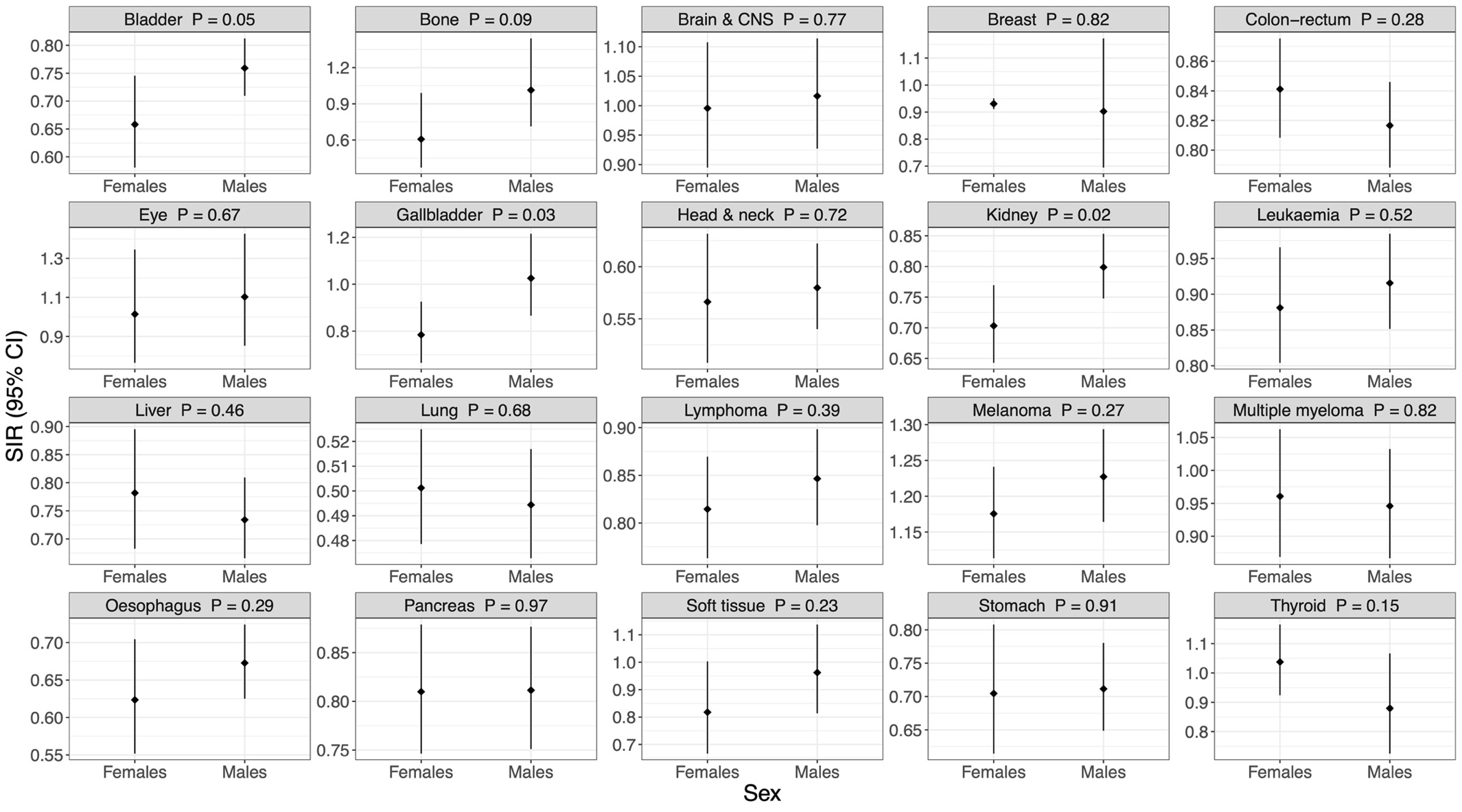

Supplement: Supplementary file 1 — Additional file 1: Tables S1–S2 and Fig. S1. Fig. S1 Standardised incidence ratios for 20 cancers by sex. [file 12916_2025_3998_MOESM1_ESM.docx]
